# The multifactorial resistance of *Pseudomonas aeruginosa*

**DOI:** 10.17179/excli2020-1249

**Published:** 2020-06-17

**Authors:** Amina Meliani

**Affiliations:** 1 Department of Biology, Faculty of Nature and Life Sciences, University Mustapha Stambouli of Mascara, 29000, Algeria

## ⁯

***Dear Editor,***

It was with great interest that we have read the review article entitled “Antibiotic resistance in *Pseudomonas aeruginosa*: mechanisms and alternative therapeutic strategies”. We truly thank Pang and colleagues for this successful research review (Pang et al., 2019[12]).

A myriad of factors involved in the *P. aeruginosa* resistance are provided in this overview. Interestingly, these multidrug resistant (MDR) bacteria have engaged an intrinsic, acquired and adaptive antibiotic resistance. In the present contribution, we are highlighting how biofilm-mediated resistance comes into play. 

Biofilm formation is often thought to represent a protective mode of growth which may enhance bacterial survival under conditions of environmental stress (Webb et al., 2003[14]). Biofilms are clusters of microbial cells that are attached to a number of different surfaces such as natural aquatic and soil environments, living tissues, medical devices or industrial or portable water piping systems (Flemming, 1995[4]).

Unsurprisingly, biofilm antibiotic susceptibility has been the subject of intense research and has been the focus of several excellent reviews (Harrison et al., 2007[7]). Biofilms render resistance to the harboring microbial cells and they are several times resistant to antimicrobials than their free living planktonic counterparts (Mah, 2012[9]). This formation is of great concern owing to it nonspecific resistance toward antibiotics, metal ions (Meliani and Bensoltane, 2016[10]), biocidal agents, protozoan grazers, desiccation and other hostile environmental conditions (Flemming and Wingender, 2010[5]).

The attached communities of microorganisms act as a protective barrier preventing the penetration of drug molecules. They secrete various inactivating and modifying enzymes which nullify the effects exerted by antimicrobials (Flemming and Wingender, 2010[5]). The increased resistance of biofilms to antibiotics allows biofilm-based infections to persist chronically in spite of antibiotic therapy (Costerton et al., 1999[3]). Bowler et al. (2012[2]) reported that the basis for biofilm-specific antibiotic resistance and tolerance is multifactorial. 

Being highly resistant within biofilm, *P. aeruginosa* has served as the prototypic model organisms for the study of antibiotics and biocides resistance. It is becoming increasingly clear that the particular antimicrobial agent, the bacterial strain, the developmental stage of the biofilm, and the biofilm growth conditions orchestrate mechanisms of resistance and tolerance.

This letter provides a proposed hypothesis focused on understanding the *Pseudomonas aeruginosa* resistance to optimize new approaches that may challenge the medical practice to fight these multidrug-resistant (MDR) bacteria. For example, the recent advances in nanotechnology can offer new prospects to develop novel formulations based on distinct types of nanoparticles (NPs) with flexible antimicrobial properties. It is also interesting to note that the structure dependent metabolic heterogeneity may also explain in part the tolerance of bacterial biofilms to these NPs. We felt that several aspects of this resistance warrant need further discussion. 

Consistent with biofilm culture studies, it has been shown that in *Pseudomonas aeruginosa* biofilms that are less than 100 µm thick, the cells that are nearest to the substratum are in anoxic zones and are slow growing. This induces an intrinsic tolerance to killing by antibiotics relative to the aerobic fast growers in the outer-biofilm layers (Walters et al., 2003[13]).

To date, extended-spectrum β lactamases (ESBLs) have the ability to hydrolyze and cause resistance to various types of β-lactam antibiotics. As an ESKAPE organism, *Pseudomonas aeruginosa* is one of the most concerning pathogens involved in antibiotic resistance. 

**Figure 1:**
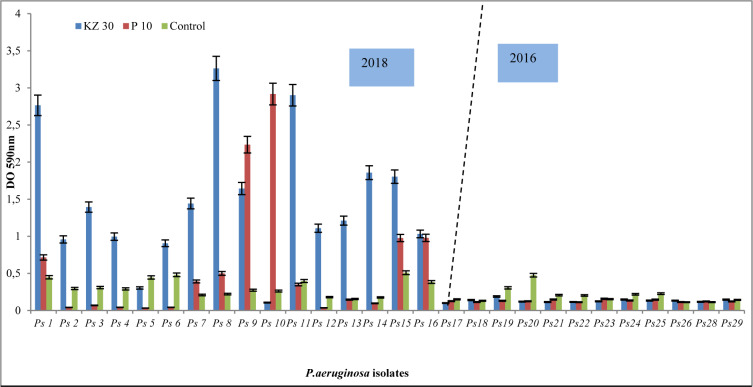
Resistance pattern of *P. aeruginosa* isolates for Cefazolin (KZ) and Penicillin (P10). Of note, an increased resistance in 2018 compared to 2016 using the frequently prescribed antibiotics of the β-lactams.

The primary outcome of a comparative study done in 2018 (Ps1…Ps16) and 2016 (Ps17…Ps29) is the increased resistance to Cefazolin (KZ30) and Penicillin (P10) (Figure 1(Fig. 1)). Many *P. aeruginosa* strains can produce different classes of ESBLs and are resistant to nearly all antibiotics, including carbapenems.

In other study, a total of 80 clinical isolates of *P. aeruginosa *were collected from different samples (March to June 2015-2016). Out of these isolates, 20 % were from urine, 30 % were from coproculture and 30 % were from cervicovaginal fluid. Generally, the isolates were found resistant following Clinical and Laboratory Standards Institute (CLSI) standards for performance and interpretation for disk diffusion susceptibility tests (NCCLS, 2004[11]). Our results clearly showed that the highest resistance levels occurred with IMP-ATM (Imipenem, Aztreonam) (60 %) while the remaining at 40 % was shown to be P-AX (Penicillin, Amoxicillin) resistant. Likewise, 30 % of isolates were resistant to AMC (Augmentin: Amoxicillin-Clavulanate), this level of resistance is relatively higher. These results are likely similar to Jones (2001[8]) study. 

The potential for *P. aeruginosa* strains to evolve antimicrobials' resistance are incompletely explored. Furthermore, the biofilm mode of growth contributes to increase this resistance.

Our previous data (Amina and Ahmed, 2017[1]) were consistent with Giwercman et al. (1991[6]) reports, in which the exposure of biofilm cells to beta-lactamase inducers triggers the production of the β-lactamase enzyme that remains associated with the biofilm.

The highlighted resistance was discussed in many scientific reports. So, the major obstacle for treatment of *P. aeruginosa* infections is the formation of biofilm and sometimes the formation of bacterial persister cells, phenotypic variants that are not genetically resistant to antibiotics but are tolerant to high concentrations of antibiotics. 

It has previously been reported that *P. aeruginosa* is thought to have acquired a resistance for other antimicrobials. In another study, we found that *P. aeruginosa *biofilms were markedly also resistant to the essential oil (Es) of *Costus *sp. The chemical composition of this oil was investigated by GC-MS (Hewlett-Packard GC-MS system (GC: 5890 séries II, MSD 5972)), it led to the identification of 45 among the 121 constituents. Linolenic acid 12, 15-Octadecatrien-1-ol, (Z,Z,Z)-) (25.03 %) and Eugenol (23.99 %) were the main components. The second major group was Bicyclo [5.2.0] nonane (6.40 %), Isogenyl acetate (4.89 %), oxide of Caryophyllene (3.02 %), α-Ionone (2.49 %) and *β*-*Eudesmol *(1.15 %) (unpublished). Consequently, *in vitro* studies have shown that the mentioned phenylpropanoid compounds did not display antibiofilm activity *vis a vis* *P. aeruginosa.*

Nevertheless, these findings have challenged the longstanding theory of essential oil-bacteria interaction since the biofilm formation was significantly correlated to oil concentrations. Additional researches are required to elucidate the direct or indirect interaction. High levels of essential oil resistance arose readily during our *in vitro* study, carried out in 2018. A significant correlation between biofilm formation and Es concentrations were obtained from both *P. aeruginosa* and* P. fluorescens* (*R^2 ^= 0,89, R^2 ^= 0,79, *respectively). Based on the data published so far, this study reveals inherent mechanisms of essential oil resistance. The key question we ask in this breakthrough study is: why the *Costus* oil did not inhibit biofilm formation? Is it because it is unable to penetrate the biofilm or it is metabolized within other pathways. 

The development of new antimicrobials is a long and winding road. Regardless of the importance of the above-mentioned results for a rewarding research area, it remains crucial to maintain a conclusion that the biofilm-mediated resistance is far more complex than initially thought.

## Conflict of interest

The author received no financial support and declares no potential conflicts of interest with respect to the authorship and/or publication of this article.
